# Supplementary material for: The genome sequence of a lauxanid fly, Meiosimyza decipiens (Loew, 1847)
Source: Wellcome Open Res. 2025 May 19;10:245. [Version 1] doi: 10.12688/wellcomeopenres.23785.1 (PMC12260490; doi:10.12688/wellcomeopenres.23785.1)
Supplement: Peer review history

# Reviewer report: Reviewer response for version 1

**DOI:** 10.21956/wellcomeopenres.26234.r124220

**Published:** 2025-07-14

**Authors:** Arun Arumugaperumal

**Affiliations:** 1 Department of Biotechnology, Rajalakshmi Engineering College, Thandalam, Chennai, Tamilnadu, 602105, India

This is the first report of the high quality genome sequence of the lauxanid fly *Meiosimyza decipiens*. The genome assembly reported here is of size 436.9 Mb and the mitochondrial genome is of size 16.39 kb. The sequencing was carried out using high end technology to arrive at a high quality genome sequence. The data are available for download from the links provided in the article.

 The link in captions of figures 2,3 &4 land on the same page. The authors can correct the link at this stage.

Are sufficient details of methods and materials provided to allow replication by others?

Yes

Is the rationale for creating the dataset(s) clearly described?

Yes

Are the datasets clearly presented in a useable and accessible format?

Yes

Are the protocols appropriate and is the work technically sound?

Yes

Reviewer Expertise:

Bioinformatics; Genomics

I confirm that I have read this submission and believe that I have an appropriate level of expertise to confirm that it is of an acceptable scientific standard.

# Reviewer report: Reviewer response for version 1

**DOI:** 10.21956/wellcomeopenres.26234.r124226

**Published:** 2025-06-12

**Authors:** Komal Kumar Bollepogu Raja

**Affiliations:** 1 Baylor College of Medicine, Houston, Texas, USA

The article by McCulloch and Crowley et al. presents the genome sequence of *Meiosimzya* *decipens* with a total length of 436.389 Megabases. The introduction is brief as not much is known about the biology of this species. The assembly metrics are impressive and meet the current standards in genome sequencing. For instance, the combined k-mer completeness is 99.64% and the BUSCO percentage is 99.64. The methodology is good and allows for reproducibility. All standard bioinformatics tools have been used by the authors. Overall, the data is well presented, high-quality and is a good genomic resource for further research.

Are sufficient details of methods and materials provided to allow replication by others?

Yes

Is the rationale for creating the dataset(s) clearly described?

Yes

Are the datasets clearly presented in a useable and accessible format?

Yes

Are the protocols appropriate and is the work technically sound?

Yes

Reviewer Expertise:

Bioinformatics, genomics, single cell genomics and Neurobiology

I confirm that I have read this submission and believe that I have an appropriate level of expertise to confirm that it is of an acceptable scientific standard.

# Reviewer report: Reviewer response for version 1

**DOI:** 10.21956/wellcomeopenres.26234.r124218

**Published:** 2025-06-05

**Authors:** Christophe Klopp

**Affiliations:** 1 INRAE, Castanet-Tolosan, France

The authors present the first genome assembly of Meiosimyza decipiens. The data types and processing steps are standard. 

 It is not clear from the material and methods if the Hi-C was used during the assembly to split haplotypes. This should be added. 

 The authors did not scaffold the second haplotype which has close to the same length as the primary one. In a pangenome perspective this would have been appreciated by downstream users. 

 It would have been interesting to have the comment of the authors on the low N50 (3.7Mb) origin for such a small genome assembled with 45x of HiFi.

Are sufficient details of methods and materials provided to allow replication by others?

Partly

Is the rationale for creating the dataset(s) clearly described?

Yes

Are the datasets clearly presented in a useable and accessible format?

Yes

Are the protocols appropriate and is the work technically sound?

Yes

Reviewer Expertise:

Genome assembly and annotation

I confirm that I have read this submission and believe that I have an appropriate level of expertise to confirm that it is of an acceptable scientific standard, however I have significant reservations, as outlined above.
